# The impact of digital transformation on corporate green governance under carbon peaking and neutrality goals: Evidence from China

**DOI:** 10.1371/journal.pone.0302432

**Published:** 2024-06-27

**Authors:** Chuyi Wang, Jitao Guo, Wei Xu, Shanyong Qin

**Affiliations:** 1 Department of Economics and Management, Qilu University of Technology (Shandong Academy of Sciences), Jinan, China; 2 School of Economics and Management, Shandong Agriculture And Engineering University, Jinan, China; 3 Shandong Women’s University, Jinan, China; Inner Mongolia University, CHINA

## Abstract

Digital transformation, as a significant shift in optimizing enterprise resource allocation and enhancing information connectivity, offers the opportunity to stimulate the endogenous dynamics of corporate green governance. Employing a sample of 3,002 listed companies in China, a fixed-effects model, and the entropy power method to formulate a green governance index system, this study examines how digital transformation affects corporate green governance concerning carbon peaking and carbon neutrality objectives. According to these findings, the implementation of the digital transformation improves corporate green governance, each unit increase in digital transformation correlates with a 1.91% enhancement in green governance. Moreover, an examination of the mechanisms shows that green governance can be promoted by addressing information asymmetry and enhancing operational efficiency. Additionally, the association between corporate green governance and digital transformation is moderated favorably by strategic aggressiveness. Furthermore, our results indicate that digital transformation contributes significantly to the advancement of green governance within enterprises located in areas with high digital financing and strong technology integration capacities. Digitalization has a stronger effect on promoting green governance for enterprises in pilot regions than in non-pilot regions in terms of carbon emission trading. This study not only assists enterprises in elucidating the developmental trajectory of digital transformation amid carbon peaking and carbon neutrality goals but also provides a reference for decision-making on how digital technology can empower corporate green governance and promote sustainable economic growth.

## 1. Introduction

As China embarks on a new stage of high-quality development, enterprises face two challenges of fostering profit growth and enhancing environmental performance. In the 75th session of the United Nations General Assembly in 2020, China proposed ambitious environmental objectives: attaining peak carbon emissions by 2030 and achieving carbon neutrality by 2060. This initiative aims to facilitate economic and social progress while promoting the green transformation of enterprises through carbon reduction strategies. Given the escalating challenges posed by global climate change and increasing constraints on resources and the environment, it has become imperative for enterprises to shift their emissions reduction strategies from a predominantly “end-of-pipe management” focus to a more proactive “source management” approach. This entails the adoption of green governance practices characterized by efficient production and technological advancement. Green governance, a new management approach that translates the concept of green development into practice, encourages enterprises to integrate green and low-carbon concepts into their entire product and service cycle, thereby establishing a long-term mechanism for sustainable development [1]. However, in the process of green governance, enterprises encounter challenges such as insufficient transformation motivation and high costs. On the one hand, some enterprises lack a strong foundation in green technology base and exhibit limited willingness to engage in green governance. According to Daily Economic News, only 8% of the 2,608 representative Chinese enterprises in southeastern coastal cities are in the green upgrading stage of their core technology installations and major operations. On the other hand, due to the high cost of comprehensive emissions management and intricate upstream and downstream interconnections within supply chains, enterprises face difficulties in implementing green management and accessing information and resources. Achieving green governance solely through policy promotion and external stakeholder pressure is challenging given multiple factors including enterprise costs and fundamentals. Therefore, enterprises must proactively seek robust measures to enhance their green governance capabilities and increase their willingness to engage in green practices. Elevating the level of green governance and fostering the dynamics of green development in enterprises have emerged as crucial factors in shaping sustainable competitive advantage and propelling China’s economy toward high-quality development.

With the rapid development of the digital economy, digital transformation has become essential for enterprises to enhance quality, increase efficiency, and drive momentum shifts. On one hand, digital transformation strategies are anticipated to facilitate cross-regional and cross-field open innovation and optimize the utilization of innovative technological resources on a broader scale. This enhances enterprises’ ability to assimilate, absorb, and re-engineer new technologies and knowledge. On the other hand, by using data modeling of industrial processes and artificial intelligence analyses during the management stage, emissions monitoring and carbon footprint tracking become feasible. Digital technology can be used as a decision-making tool for “green product design” and energy saving initiatives, facilitating systematic whole-life-cycle management of enterprises and creating opportunities for green governance. However, digital transformation requires significant resources, and initial investments may not yield quick returns, potentially leading to resource crowding and increased financial risk [2]. Can digital transformation break through the technological and cost constraints to advance the “dual-carbon” goal and drive corporate green governance? What mechanisms underlie the impact of digital transformation on green governance? Exploring these questions will not only reveal the intrinsic drivers and realization paths of enhancing corporate green governance but also provide intellectual support and serve as a reference for decision making when implementing digital technologies.

This study explores the relationship and potential mechanisms between digital transformation and corporate green governance using data from listed enterprises from 2012 to 2021. It aims to make several marginal contributions: First, while existing literature extensively examines the influence of internal and external stakeholders on corporate green governance, such as management’s environmental awareness, green investor participation, public recognition, and government environmental policies, it overlooks the governance effect and driving role of digitalization. This study integrates digital transformation and green governance into a unified framework, expanding the scope of the research and invigorating the dynamics of corporate green governance. Second, while some scholars argue that strategic change is crucial in the digitalization process and assert that strategic orientation constitutes a vital foundation for the direction of corporate green governance, they seldom explore the mechanisms linking these elements at the strategic level. This study focuses on the strategic level of enterprises and analyses its impact on the relationship between digital transformation and green governance from the perspective of strategic aggressiveness. By enhancing our understanding of the mechanisms through which digital transformation affects green governance, this study provides a theoretical foundation for future corporate decision making and strategic planning. Finally, this study analyzes the digital financial development landscape, technology integration capacity, and the background of carbon emissions trading pilot programs to explore the specific context in which digital transformation promotes green governance. It not only provides a practical reference for releasing digital dividends and deepening the green application of digital technology but also offers useful insights for the government to enhance the vitality of green governance and promote sustainable economic development.

## 2. Literature review

Corporate green governance refers to integration of organizational and environmental objectives by enterprises, aiming for sustainable development, and fulfilling social responsibility through innovative technologies, methods, and models [3]. Scholarly research has extensively explored strategies to enhance corporate green governance, which can be broadly categorized into three approaches: strengthening corporate environment-oriented operations, responding to stakeholders’ green value propositions, and digitalization and intelligence. From a corporate environment-oriented management perspective, studies have found that executive green cognition, green innovation, and environmental management control positively influence green governance. Liu and Cao [4] argue that executives with environmental cognition can grasp potential market opportunities, incorporate environmental issues into corporate strategic objectives, and guide firms in applying their limited resources and capabilities to green governance activities to enhance corporate sustainability performance. According to Luan *et al*. [5], green innovation positively impacts energy-consuming enterprises’ performance in reducing carbon emissions. Carbon emissions can be decreased by raising awareness of green innovation and using technologies such as carbon capture and cleaner production processes to enhance the effectiveness of environmental governance. Regarding stakeholders’ green value propositions, numerous scholars argue that relevant initiatives from the government, green investors, the public, and the media play significant roles in influencing corporate green governance. As highlighted by Yu *et al*. [6], voluntary environmental regulation policies incentivize enterprises to proactively disclose environmental information. Such disclosures facilitate peer-to-peer comparisons and monitoring, mitigate environmental management risks, and foster green corporate governance. Geng *et al*. [7] and Chi and Yang [8] examine the differences and synergies in the impacts of the public and media on corporate green governance and find that both the public environmental demands and media coverage boost corporate green governance. According to Lei and Yu [9], companies with green investors are more likely to adopt green initiatives, increase green spending, and enhance green governance performance, confirming the role of stakeholders’ green orientation as an external facilitator of corporate green governance. Regarding digitalization and intelligence, scholars contend that digital technologies may serve as technical and decision-making aids when viewed through the lens of digitalization and intelligence. Adopting the dual theory and fuzzy-set qualitative comparative analysis (fsQCA) approach, Yu and Sui [10] underline the need to combine digital instrumental factors and corporate digital responsibility to improve firms’ economic, environmental, and social performance. Guo *et al*. [11] argue that improved digital infrastructure facilitates online “paperless” working for enterprises, which reduces resource consumption and contributes to the green transition. Shen and Zhang [12] argue that the “technological dividend” brought about by the evolution of artificial intelligence introduces a new paradigm for enterprises to achieve green governance. This significantly reduces enterprises’ pollution emission intensity through intelligent governance, which is crucial for front-end control, process management, and end-end governance.

Many scholars have conducted useful explorations of digital transformation and corporate green practices; however, some debates have arisen regarding its impact. Many academics believe that digital transformation provides new opportunities for the procurement, production, and emissions phases of green governance. Lai *et al*. [13] conclude, through a case study, that the creation of a platform for supply chain collaboration and pooling of idle resources can help digital transformation create a green supply chain, which can lead to the formation of communities of interest among enterprises and enhance corporate willingness toward green governance. Lin and Xie [14] find that digital transformation helps firms accurately predict market demand, strategically allocate innovation resources to key areas, improve the conversion and utilization efficiency of resources, and create a competitive advantage for the green governance of firms at the production stage. Wang *et al*. [15] empirically test the emission reduction effect of digital transformation, which mainly comes from source prevention and control, rather than end-of-pipe management. Some academics have presented an alternative viewpoint, contending that there are few energy-saving advantages to digital transformation. According to this view, the excessive use of technology might lead to an information overload and an imbalance in the distribution of resources [16], which will stifle green innovation and leave businesses with insufficient kinetic energy for green governance [17].

Along with the introduction and implementation of the “dual-carbon” goal, corporate green governance presents the new features of source governance and whole-life-cycle management. Considering carbon peaking and neutrality, enterprises must transition from end-to-end governance to whole-process governance to maintain a green and low-carbon presence throughout the lifecycle of goods and services [18]. In the practice of green governance, a digital transformation strategy enables enterprises to achieve both source and end green innovation [19]. However, source control is more proactive and long-term in green governance, and digital transformation can achieve energy consumption control and pollution monitoring throughout the product life cycle of products while significantly improving enterprises’ productivity [20]. Thus, digital transformation serves as a greater driving force for source prevention and cleaner production in green governance. Therefore, it is important to examine the connection between digital transformation and green governance within the framework of the dual carbon targets.

In general, the extant literature centers on strategies to enhance green governance and the facilitating effects of digital transformation on environmental sustainability within enterprises, a subject that has been extensively investigated and has generated valuable outcomes. Nevertheless, little attention has been paid to how the digital revolution has affected corporate green governance and few analyses have been conducted within the framework of carbon peaking and neutrality goals. This paper delves into the emerging characteristics of green governance within the framework of the “dual-carbon” goal. It investigates the logic of value creation through corporate digitalization in green governance. This not only strengthens the logical evolution of digital transformation to support the low-carbon and environmentally friendly development of companies but also provides a useful guide for the sustainable growth of China’s economy throughout the carbon peak and carbon neutrality phases.

## 3. Theoretical analysis and research hypotheses

### 3.1 Digital transformation and corporate green governance

As digital transformation progresses, it strengthens connectivity both within enterprises and between upstream and downstream partners, and key resources such as information and technology gradually accumulate. This is beneficial for enterprises to strengthen internal controls, improve technological innovation, and collaborate in the supply chain, enabling their green activities to achieve transparency, efficiency, and synergy, thereby impacting corporate green governance.

On the one hand, the digital revolution supports management in making better decisions about green governance and enhances the caliber of internal control. Digital technology can be used to apply internal controls to every facet of a company’s operations, enabling real-time tracking, thorough monitoring, and dynamic modification [21], thus opening paths for strengthening internal monitoring mechanisms, implementing efficient green management, and improving corporate green governance. In addition, the digital revolution makes it possible to visualize business outcomes, which can weaken managers’ discretionary power by enhancing the digitalization of management processes and reducing room for speculation by managers, thus enabling shareholders to supervise management effectively [22, 23]. Consequently, shareholders can effectively supervise management, grasp the green governance of enterprises, and encourage management to implement sustainable development strategies in alignment with green management concepts. On the other hand, digital transformation can stimulate innovation in R&D and improve corporate green governance. In terms of innovation efficiency, based on digital technology, businesses can shorten the R&D cycle by building a product “digital twin” model, lowering the cost of trial and error associated with green product R&D and raising the bar for corporate green governance, all of which will accelerate the process of developing green, differentiated products [19]. Regarding resources for innovation, digitalization gives enterprises the ability to innovate collaboratively with innovation bodies such as universities and research institutes, overcoming geographical and temporal limitations. Through this process, enterprises can integrate the R&D resources required for green innovation on a wider scale, master the core technology and development trends in green innovation, and provide technical support for green governance.

Digital transformation strengthens collaboration and interaction between upstream and downstream supply chain enterprises, thereby enhancing corporate green governance. Green governance has the characteristic of linkage, and in order to respond to the “dual-carbon” strategy and resist market risks, enterprises can jointly promote green actions with more enterprises within the supply chain. With the continued advancement of digital transformation, businesses have established digital platforms such as the Internet of Things and intelligent integration systems. Information, capital, technology, and other factors have all fully flowed through the supply chain, continuously improving the efficiency of resource allocation [24], and leading to a highly efficient connection between resources and information sharing. This provides significant convenience for enterprises, enabling them to coordinate with upstream and downstream enterprises in terms of green production and sales, and increases the accuracy of green demand forecasting. Based on this, enterprises can not only screen green suppliers by comparing a large amount of information and reduce pollution emissions by checking products at the source, but also build green supply chains in close collaboration with upstream and downstream suppliers to enhance their green governance capabilities. Furthermore, the benefits of digital transformation in terms of information and resource availability push greening of the downstream demand side, which in turn promotes green governance upstream. Additionally, upstream greening raises the standards for downstream enterprises’ production processes, accelerating the supply chain’s capacity for green governance. Based on the aforementioned studies, we propose the following hypothesis.

H1: Digital transformation can improve corporate green governance.

### 3.2 The path of digital transformation to corporate green governance

#### 3.2.1 Digital transformation promotes corporate green governance by mitigating the level of information asymmetry

The adaptability and permeability of digital technology, as observed by external stakeholders, provide “zero distance” between enterprises and stakeholders. With the increasing use of digital technology, interactive systems of enterprise information can be created to help external parties understand the true state of an enterprise’s operations and production. This will also help enterprises understand the green needs of their partners and obtain access to state-of-the-art industry information. This alleviates information asymmetry, and ultimately forms a transparent space for reciprocal values. Under the role of the information asymmetry mechanism, the effectiveness of green regulation is greatly enhanced, the “strategic response” and “greenwashing” behaviors of enterprises are gradually reduced, and ongoing improvements are made to the information disclosure mechanism. Enterprises will take the lead in projecting a green image and sharing environmental data to communicate positively with the outside world, which not only lays the foundation for obtaining resources for green action, but also enhances their willingness toward corporate green governance and responds to the requirements of dual-carbon goals.

From an internal perspective, digital transformation may decrease the cost of information disclosure through the openness of internal processes; accelerate the distribution and feedback of information across departments [25], and enable the sharing of green information among a company’s manufacturing, management, and R&D departments, thereby alleviating information imbalance. Additionally, digital mining technology involves digital archiving and data processing, which can provide real and reliable data for corporate management [26] and reduce distortions in the information transmission process in each department. An increase in information transparency is conducive to realizing timely monitoring and correction of green management, which makes corporate green governance scientific, efficient, and promotes it. Based on the aforementioned studies, we propose the following hypothesis.

H2: Digital transformation promotes corporate green governance by alleviating information asymmetry.

#### 3.2.2 Digital transformation facilitates corporate green governance by improving enterprises’ operational efficiency

By combining multiple technologies, such as data mining, operational management, and decision support, digital transformation can improve interdepartmental communication efficiency, reduce coordination costs [27], and create a methodical operation model for smart and effective operation management. Additionally, the integration of digital technology into enterprise operations management has led enterprises to advance to a lean management stage [28]. As digital transformation continues, enterprises may utilize technological instruments such as sensors, RFID, and GPS to obtain the time trajectory and spatial position data of production elements. This allows them to move from manual and empirical production to precise and standardized production [29]. This lays the foundation for enterprises to rationally allocate production factors and flexibly release production capacity, which helps them to make better use of their assets within the constraints of available resources, thereby boosting operational effectiveness.

Operational efficiency is an important indicator of asset operations. In the pursuit of enhanced operational efficiency, companies reduce the use of industrial equipment with high energy and emission levels and actively carry out equipment renovation and upgrading. This will control the generation of pollutants at the source, enable enterprises to be green at the front end [30, 31], and promote green governance while contributing to carbon peaking and neutrality strategies. In addition, higher operational efficiency signifies rapid turnover and proper coordination of enterprise resources, which not only strengthens the connection of resources in the supply chain to facilitate green production and sales but also mitigates potential resource conflicts between digital transformation and green governance, creating favorable resource conditions for corporate green governance. Based on the aforementioned analyses, we propose the following hypotheses:

H3: Digital transformation promotes corporate green governance by enhancing corporate operational efficiency.

### 3.3 Moderating effects of strategic aggressiveness

Digital transformation can create opportunities for promoting corporate green governance, but it also implies changes in the mode of production, business processes, and many other aspects, during which enterprises face the challenge of breaking through organizational inertia and forming new practices. This requires the promotion of top management and the overall deployment of resources, and calls for top-down planning and implementation at a strategic level; otherwise, the difficulty and complexity of enterprise digital transformation will increase exponentially. Thus, strategic choices influence how digital transformation affects corporate green governance.

Strategic aggressiveness reflects an organization’s propensity to make strategic decisions linked to resource allocation and future development paths and affects how corporate green governance and digital transformation interact. First, strategic aggressiveness amplifies the influence of digitalization on green governance through improved operational efficiency. Enterprises that exhibit higher levels of strategic aggressiveness are more committed to attaining rapid growth through market expansion, can use resources efficiently and in a timely manner in response to market changes, and are eager to produce new goods [32]. These advantages help enterprises improve their operational management efficiency to enhancing the operational efficiency path of digital transformation and contributing to the rapid turnover of green governance resources and accurate docking. Second, for enterprises with a more aggressive strategy, an atmosphere of restructuring and change fills the organization, and employees are more receptive to emerging digital technologies. The promotion of digital transformation can gather more support and cooperation, making it easier for enterprises to overcome R&D inertia and implementation resistance in the transformation process. This facilitates the application of digital technology to green management and innovation, creating a favorable atmosphere for digital transformation to promote green governance. Third, according to resource dependence theory, enterprises need to obtain key resources from external stakeholders. For enterprises adopting radical strategies, management pays more attention to establishing good relationships with stakeholders and actively fulfilling their social responsibilities [33]. To better support digital technology and corporate green governance during the digitalization process, these enterprises should work together to improve communication channels between downstream and upstream enterprises and form a green image in green production and sales. Based on this analysis, we propose the following hypotheses:

H4: Strategic aggressiveness positively moderates the relationship between digital transformation and corporate governance.

## 4. Study design

### 4.1 Sample selection and data sources

Since the 18th Party Congress elevated the digital economy as a national strategy in 2012, China implemented a series of policies related to digital technologies. Influenced by these policies, enterprises have begun to explore digital technology in depth and have carried out large-scale digital transformations. Based on the above background and data availability, this study uses A-share listed companies from 2012 to 2021 as the research sample to empirically investigate the link and mechanism between digital transformation and corporate green governance. The primary data sources are firms’ annual reports, the Chinese Research Data Services database (CNRDS), and the China Stock Market and Accounting Research database (CSMAR). The data were processed based on the following criteria: (1) exclusion of ST and PT companies; (2) exclusion of samples from the financial industry; (3) exclusion of enterprises from research that lacked pertinent data in its records; (4) to reduce the impact of extreme results, every sample was shrink-tailed at the one percent threshold; and (5) standardization of all continuous variables, resulting in 22,211 observations.

### 4.2 Regression model settings

To test H1, model (1) was built to evaluate the effects of digital transformation on green governance.


$$Greengov_{i,t}=\alpha_{0}+\alpha_{1}\;Digital_{i,t}+\alpha_{2}\;Control_{i,t}+\sum \;Year+\sum \;Firm+\varepsilon_{i,t}$$ (1)

Where *Greengov*_*i*,*t*_ and *Digital*_*i*,*t*_ represent the level of corporate green governance and the extent of enterprise i’s digital transformation in year t. *Control*_*i*,*t*_ signifies the control variables, and ∑*Year*, ∑*Firm* and ε_*i*,*t*_ stand for the year fixed effect, the individual fixed effect, and the random disturbance term, respectively.

To test H2 and H3, models (1) to (3) were used to evaluate the impact of digital transformation on corporate green governance.


$$Mediator_{i,t}=\gamma_{0}+\gamma_{1}\;Digital_{i,t}+\gamma_{2}\;Control_{i,t}+\sum \;Year+\sum \;Firm+\varepsilon_{i,t}$$ (2)

$$Greengov_{i,t}=\beta_{0}+\beta_{1}\;Digital_{i,t}+\beta_{2}\;Mediator_{i,t}+\beta_{3}\;Control_{i,t}+\sum \;Year+\sum \;Firm+\varepsilon_{i,t}$$ (3)

Where *Mediator*_*i*,*t*_ represents the mediating variables information asymmetry degree and operational efficiency.

To test H4, model (4) was used to test the moderating effect of strategic aggressiveness on the link between digitalization and corporate green governance.


$$Greengov_{i,t}=\theta_{0}+\theta_{1}\;Digital_{i,t}+\theta_{2}\;Digital_{i,t}\times \;{\text{Strategy}}_{i,t}+\theta_{3}\;{\text{Strategy}}_{i,t}+\theta_{4}\;Control_{i,t}+\sum \;Year+\sum \;Firm+\varepsilon_{i,t}$$ (4)

Where Strategy_*i*,*t*_ denotes the strategic aggressiveness of enterprise i in year t.

### 4.3 Variable selection

#### 4.3.1 Explanatory variable

Digital transformation (digital). Based on the findings of Wu *et al*. [34], text analysis is one way to evaluate digital transformation. Five dimensions were included in the first step of building the digital transformation feature word map: cloud computing, artificial intelligence, big data, blockchain, and digital technology applications. Next, a text analysis of enterprise annual reports was conducted using Python, and the total word frequency was statistically derived. Finally, considering the evident right-skewed characteristics of the data, the total word frequency underwent a logarithmic transformation after the addition of one to obtain the final index of enterprise digital transformation.

#### 4.3.2 Explained variable

Corporate green governance (Greengov). In the context of the “dual-carbon” goal, enterprises should integrate the green concept into the whole process of governance. This requires enterprises not only to consider carbon emissions governance but also to form a green culture, consciously implement green management, strictly enforce green emissions, provide timely disclosure of green information, and carry out green supervision throughout all production and operational processes. Therefore, this study builds on the research conducted by Huang *et al*. [35] to construct an indicator system for corporate green governance based on the five dimensions listed in Table 1. For qualitative indicators, a content analysis methodology was employed to score the qualitative indicators according to their level of detail. For example, for the indicator of “special environmental protection activities”, if no special environmental protection activities were disclosed or carried out, the value was 0; if special environmental protection activities were mentioned, the value was 1; and if the specific content of special environmental protection activities was disclosed, the value was 2. For the quantitative indices, the natural logarithm was used to reduce the degree of data discrepancy. The remaining indicators were qualitative, whereas green technological innovation and investment were quantitative. Based on this, the weight of each corporate green governance evaluation index is determined using the entropy weighting approach, which is then used to generate a final corporate green governance score.

**Table 1:** Corporate green governance indicator system.

Principal index	Secondary Index	Principal index	Secondary Index
Green environment	Environmental concept	Green management	Green technology innovation
	Environmental goals		Green Investment
	Environmental education and training		Cleaner production
	Environmental actions		Three simultaneous system
	Environmental honors and awards		Environmental management system
Green emissions	Pollutant emission compliance	Green information	Environmental information disclosure in annual report
	Emission reduction and management of waste gas		Social responsibility report publication of environmental information
	Wastewater emission reduction management		Environmental report information disclosure
	Dust and fume management		Environmental information Emergency response mechanism
	Utilization and disposal of solid waste		
	Light pollution, radiation, noise, and other governance issues		
Green regulation and certification	Environmental emergencies		
	Environmental violations		
	Environmental petitions		
	ISO14001 certification		
	ISO9001 certification		

#### 4.3.3 Mediating variables

Degree of information asymmetry (analysts). Drawing on Shen and Tan [36], the information asymmetry of an enterprise was quantified by the count of analysts tracked, incremented by one, and then subjected to natural logarithm transformation. A rise in analysts’ following corresponds to a reduction in the degree of knowledge asymmetry.

Operational efficiency (efficiency). Drawing on Zhang [37], an enterprise’s operational efficiency was gauged by the cost rate incurred during the sales period, namely, (sales cost + management cost + financial cost)/ (operating income).

#### 4.3.4 Moderating variable

Strategic Aggressiveness (strategy). According to Bentley *et al*. [38], indicators of corporate strategic aggressiveness are constructed from six dimensions: R&D expenses as a proportion of sales income; employee-to-sales ratio; historical growth rate of sales revenue; proportion of selling and administrative costs to sales revenue; fluctuations in employee numbers; and the percentage of fixed total assets fixed. These indicators reflect the characteristics of an enterprise such as innovation tendencies, market development tendencies, and organizational stability. Subsequently, the six variables were averaged over the preceding five years. For the initial five variables, each sample was categorized into five groups from small to large, attributing a score of 0 to the smallest group and 4 to the largest. Conversely, for the sixth variable, the grouping was inverted, assigning a score of four to the smallest group and zero to the largest group. By adding the scores of the six variables to each sample, a composite score was obtained to measure corporate strategic aggressiveness, with a range of 0 to 24 points. Higher scores imply a more aggressive strategy, whereas lower scores indicate a more conservative strategy.

#### 4.3.5 Control variables

Following existing studies [35], we introduce a series of control variables related to green governance, including the asset-liability ratio (Lev), nature of property rights (Soe), board size (Board), years of listing (Listage), institutional investor shareholding (Inst), operational activities’ net cash flow (Ncf), proficiency (Roa), and growth (Growth). Table 2 summarizes these variables.

**Table 2:** Variable definitions.

Variable type	Name	Variable	Definition
Explained variable	Corporate green governance	Greengov	Utilize the entropy weighting approach to determine the weight of each green governance indicator and calculate the final score
Explanatory variable	Digital transformation	Digital	Get the total word frequency by text analysis method, plus 1 to take the natural logarithm
Mediating variable	Level of information asymmetry	Analyst	The count of analysts tracked add one to get the logarithm
	Operational efficiency	Efficiency	(Selling expenses + administrative expenses + financial expenses) / operating income
Moderating variable	Strategic aggressiveness	Strategy	Total the scores of the 6 indicators. The higher the score, the more aggressive the strategy is
Control variable	Asset gearing ratio	Lev	Total liabilities at year’s end / Total assets at year’s end
	Nature of ownership	Soe	State-owned enterprises are assigned the value of 1, while others receive a value of 0
	Board size	Board	The quantity of directors on the board takes logarithm
	Years of listing	Listage	Natural logarithm of the duration of the listed enterprise has been publicly traded.
	Institutional investor shareholding	Inst	Institutional investors’ shareholding as a proportion of circulating shares
	Net cash flow from operating activities	Ncf	Net cash flow from operating activities divided by total assets at the period’s conclusion
	Profitability	Roa	Net profit / average balance of total assets
	Growth	Growth	The rate of growth in operating income

## 5. Results

### 5.1 Descriptive statistics

Table 3 displays the descriptive statistics before the core variables were standardized, revealing disparities in green governance across diverse enterprises, as seen by the greatest value of 0.7397 and the lowest value of 0.0003 for corporate green governance (Greengov). With a mean score of 0.1645 and median of 0.1068, it is clear that over half of the enterprises practice poor green governance. With a median value of 1.0986 and an average of 1.4591, digital transformation (Digital) indicates that some enterprises were still in the early phases of this process.

**Table 3:** Descriptive statistics.

Variable	Mean	Median	Std. Dev.	Min	Max	Obs.
Greengov	0.1645	0.1068	0.1762	0.0003	0.7397	22,211
Digital	1.4591	1.0986	1.3997	0.0000	5.0999	22,211
Lev	0.4319	0.4217	0.2088	0.0586	0.9426	22,211
Soe	0.3091	0.0000	0.4621	0.0000	1.0000	22,211
Board	2.1169	2.1972	0.1955	1.6094	2.6391	22,211
Listage	2.8776	2.9269	0.3401	1.8326	3.5065	22,211
Inst	0.4381	0.4533	0.2475	0.0034	0.9132	22,211
Ncf	0.0476	0.0469	0.0686	-0.1617	0.2455	22,211
Roa	0.0405	0.0402	0.0730	-0.3042	0.2354	22,211
Growth	0.1891	0.1121	0.4756	-0.6081	3.2243	22,211

### 5.2 Baseline regression results

The results are summarized in Table 4. As shown in Column (1), the coefficient of the explanatory variable (Digital) is positive at the 1% level. The control variables were not included in the analysis. Column (2) presents the results of the regression analysis using the included control variables. The coefficient was 0.0191 and the significance level was set at 1%. It is clear that, even when control factors are included, there is a consistently substantial positive link between digital revolution and green governance, suggesting that digital transformation can support corporate green governance. Therefore, H1 is confirmed.

**Table 4:** Baseline model test.

Variable	(1)	(2)
	Greengov	Greengov
Digital	0.0239***	0.0191***
	(0.009)	(0.007)
Lev		0.0075
		(0.009)
Soe		-0.0014
		(0.006)
Board		-0.0132
		(0.010)
Listage		0.0536
		(0.038)
Inst		0.0241**
		(0.010)
Ncf		0.0040
		(0.005)
Roa		0.0419***
		(0.008)
Growth		-0.0154**
		(0.006)
Constant	0.1188***	0.0569***
	(0.003)	(0.020)
Year	YES	YES
Firm	YES	YES
Observations R^2^	22,211 0.15	22,211 0.15

Note: Robust standard errors are reported in parentheses, ***, ** and * indicates the significance at the 1%, 5%, and 10% levels, respectively, as follows.

### 5.3 Robustness test

#### 5.3.1 Substitution of explained variable and explanatory variable

The role of digitalization in green governance may be affected by the selection of indicators for core variables. To avoid interference from this effect, we replace the explanatory and interpretative variables separately. Columns (1)–(3) in Table 5 present the findings after replacing the core variables. First, an environmental score of “E” for an enterprise in the Huazheng ESG scoring system was selected as the explanatory variable. Given that performance indicators are rated using environmental scores such as the carbon emissions and energy consumption intensity of enterprises, they can reflect the effectiveness of corporate green governance. Column (1) of Table 5 presents the findings. The coefficient of the explanatory variables’ coefficient is 0.0030, indicating significance at the 5% level. Second, according to Huang *et al*.’s (2022) study, corporate green governance is measured using the ratio of “corporate green governance” to “operating earnings.” Column (2) shows that the regression coefficient for digital evolution is positive. Finally, based on Lin and Xie [27], a composite index of digital transformation is created using principal component analysis from the three dimensions of digital strategy, digital inputs, and digital outputs. Column (3) of Table 5 displays the results, in which the regression coefficients are still significant and positive at the 1% level.

**Table 5:** Robustness test.

	(1)	(2)	(3)	(4)	(5)	(6)	(7)
	Greengov	Greengov	Greengov	Digital	Greengov	Greengov	L.Greengov
Digital	0.0030**	0.0001**	0.0555***		0.0168*	0.0215***	0.0180***
	(0.001)	(0.000)	(0.0177)		(0.0096)	(0.0066)	(0.007)
IV1				0.6713*** (0.0163)			
IV2				0.3087*** (0.0098)			
LM statistic				2002.049 [0.000]			
Wald F statistic Hansen J statistic				4587.910 {19.93} 0.096 [0.7566]			
Control	YES	YES	YES	YES	YES	YES	
Year	YES	YES	YES	YES	YES	YES	
Firm	YES	YES	YES	YES	YES	YES	
Year × Industry						YES	
Year ×Province						YES	
Constant	0.4314***	0.0030***	0.0655***	-0.0065	0.1442***	0.0856***	0.0824***
	(0.016)	(0.001)	(0.0212)	(0.0239)	(0.0217)	(0.0263)	(0.023)
Observations	21,462	22,210	20842	19120	19120	22211	19,120
R^2^	0.05	0.14	0.164	0.50	0.01	0.16	0.11

Note: p-values in [ ] and critical values at the 10 per cent level of the Stock-Yogo weak identification test in { }.

#### 5.3.2 Endogeneity tests

*Instrumental variable method*. To mitigate the potential endogeneity problem, we used the two-stage least squares method for the instrumental variable regression. Following the approach outlined by Lin and Xie [14] and Qi and Song [39], instrumental variables for digital transformation were selected. Specifically, the first-order lag term of digital transformation (IV2) and the mean value of digital transformation within the same province and industry (IV1) were chosen as instrumental variables. These variables satisfied the correlation with digital transformation and were not affected by current digital transformation, thereby satisfying the correlation and exogeneity requirements. Columns (4) and (5) of Table 5 display the findings of the first- and second-stage regression analyses, respectively. The Kleibergen-Paap rk LM statistic is statistically significant at the 1% level, thus rejecting the original hypothesis of the under-identification of instrumental variables. The Cragg-Donald Wald F-statistic exceeds the critical value at the 10% level, indicating that there was no weak instrumental variable problem. The Hansen J statistic, with a p-value of 0.7958, suggests that there is no overidentification problem with the instrumental variables. In Column (4), the regression coefficients of both instrumental variables are significantly positive at the 1% level, demonstrating consistency with the correlation. In Column (5), the regression coefficient for digital transformation is significantly positive at the 10% level, confirming that this study’s conclusions remain valid after controlling for interference from potential endogeneity problems.

*Adding the interaction terms to the research model*. There may be some omitted variables in the model; therefore, we added the cross-multiplier terms Year × Industry and Year × Province to the research model. Column (6) of Table 5 shows that the regression coefficients remain significant at the 1% level.

*One-period lag test*. The explanatory variable was one period, to account for any potential time-lag effects of digital change on green governance. Column (7) presents the results of the analysis. The robustness of the results is evident from the persistence of positive and statistically significant coefficients of the explanatory factor.

### 5.4 Mechanism tests

#### 5.4.1 Tests of mediating effects

Table 6 presents the results of impact path tests. Column (1) shows the regression results for the explanatory variables of the degree of information asymmetry. The significance of the coefficient is confirmed, indicating that information transparency improves through digital transformation.

**Table 6:** Influence mechanism test.

Variable	Mediation effect test of the degree of information asymmetry		Mediation effect test of operational efficiency		Moderating effect test of strategic aggressiveness
	(1) Analyst	(2) Greengov	(3) Efficiency	(4) Greengov	(5) Greengov
Digital	0.1150***	0.0153**	-0.0337***	0.0181***	0.0270***
	(0.014)	(0.007)	(0.008)	(0.007)	(0.008)
Analyst		0.0332***			
		(0.005)			
Efficiency				-0.0311***	
				(0.009)	
Digital×Strategy					0.0760***
					(0.026)
Control	YES	YES	YES	YES	YES
Year	YES	YES	YES	YES	YES
Firm	YES	YES	YES	YES	YES
Constant	-0.0379	0.0581***	0.4542***	0.0706***	0.0730**
	(0.043)	(0.020)	(0.024)	(0.020)	(0.032)
Observations	22,211	22,211	22,122	22,122	15,089
R^2^	0.18	0.16	0.18	0.16	0.14

Second, the extent of the information imbalance was included in the benchmark regression model, as indicated in the second column. The coefficient of information asymmetry (Analyst) was significantly positive, demonstrating that increasing information transparency increases the need for green governance. Additionally, the digital transformation coefficient (Digital) was smaller than that in the benchmark regression, indicating that the path of information transparency was established. Thus, H2 was verified.

Similarly, digital transformation regresses operational efficiency, as shown in Column (3). The digital transformation (Digital) coefficient is significantly negative, suggesting that digitalization leads to a reduction in the expense ratio during sales and improves operational efficiency. Second, operational efficiency was included in the benchmark regression model, as shown in Column (4), where this coefficient stands at -0.0311 and statistical significance is at the 1% level. This finding indicates that enhancing operational efficiency fosters corporate green governance. Furthermore, the coefficient of the explanatory variable (Digital) was lower than that in the benchmark regression, suggesting that the pathways through which operational efficiency has an impact are well established. Digital transformation can improve green corporate governance by enhancing operational efficiency. Therefore, H3 was verified.

#### 5.4.2 Moderating effect test

Column (5) of Table 6 presents the results of the moderating impact analysis. The interaction coefficient of digital transformation and strategy aggressiveness was positive at the 1% significance level. This outcome demonstrates that the interaction between digital transformation and corporate green governance is favorably moderated by strategy aggressiveness, which aligns with the projected implications of H4.

## 6. Further research

### 6.1 Further analysis based on the digital financial development environment

As an emerging financial tool, digital finance enhances the mobility efficiency of information and resources, and provides a robust technological foundation for digital transformation [40]. Consequently, the effects of digital transition on green governance may vary across diverse digital finance development environments. We employ the Peking University Digital Financial Inclusion Index to assess the state of digital finance at the provincial level. If the index surpasses its median, indicating a superior digital financial development environment for the enterprise, the dummy variable is set to 1; otherwise, it is set to 0. The findings presented in Columns (1) and (2) of Table 7 suggest that enterprises situated in areas with advanced digital financial growth have substantially positive coefficients for digitalization (Digital). However, the contribution of digital transformation to corporate environmental governance is not apparent in enterprises located in areas with low digital financial growth.

**Table 7:** Further analyses.

Variable	(1) High level of digital financial development	(2) Low level of digital financial development	(3) Strong technological integration capability	(4) Weak technological integration capability	(5) Carbon emission trading pilot regions	(6) Carbon emissions trading non-pilot regions
Digital	0.0213**	0.0153	0.0399***	0.0114	0.0214**	0.0194**
	(0.008)	(0.011)	(0.012)	(0.013)	(0.010)	(0.0086)
Control	YES	YES	YES	YES	YES	YES
Year	YES	YES	YES	YES	YES	YES
Firm	YES	YES	YES	YES	YES	YES
Constant	0.0662***	0.0350	0.1197**	0.1760***	-0.0051	0.0857***
	(0.023)	(0.041)	(0.060)	(0.066)	(0.0398)	(0.0278)
Observations	13,833	8,373	7,415	7,404	8435	13771
R^2^	0.15	0.17	0.15	0.15	0.14	0.16

One possible reason is that digital finance can effectively alleviate the information asymmetry problem on the supply and demand sides of traditional financial resources and realize the precise matching of information between subjects [41]. This helps mitigate information asymmetry between capital providers and enterprises, allowing the information effect of digital transformation to be effectively leveraged to lower the cost of financing green governance activities and stimulate green investment. However, in areas with lower levels, there is less potential to improve operational performance and external assistance from the digital finance sector for digital transformation [42]. This results in a decreased rate of upstream and downstream resource integration, consequently affecting the green production and sales chain, and impeding the full demonstration of the effect of digital transformation on environmental governance. Thus, digitalization has a greater incentive effect on green governance for enterprises in regions with advanced digital finance development.

### 6.2 Further analysis based on technology integration capability

Technology integration capability refers to an enterprise’s capacity to digest and integrate technological resources through technology evaluation, selection, and refinement under uncertain conditions [43]. With improvements in technological integration capability, enterprise innovation resources are gathered and innovation efficiency is improved. Digital transformation can drive enterprise innovation and enhance green governance, but the effects may differ based on an organization’s technological integration capabilities. We quantified the technological integration capability using the percentage of R&D professionals based on pertinent research. When the technological integration capability was greater than its median, it demonstrated that the enterprise’s technology integration capability was stronger, and the dummy variable was set to 1; otherwise, it was set to 0. The findings presented in columns (3) and (4) of Table 7 indicate that the coefficient of digital transformation (Digital) is significant when enterprises have a better capacity for technology integration. For enterprises with limited technological integration skills, the coefficient was not significant. This signifies that digital transformation strongly encourages green governance in enterprises with a higher capacity for technology integration.

A likely explanation is that enterprises with stronger technological integration capabilities have more technological resources and a stronger innovative edge, and the digital transformation process is more efficient in transforming information, knowledge, and technology into internal innovations. These enterprises purchase new environmental equipment, hire experts to assess the feasibility of green technologies, and employ high-quality human capital. Furthermore, stronger technological integration capability, a major advantage for enterprises in developing new technologies, enables digital transformation to drive R&D and innovation, which, in turn, provides technical support for green governance and accelerates the process of digital transformation for green governance. Enterprises with limited technological integration capabilities face challenges in applying technologies across diverse areas. Digital technology assumes a restricted role in the realm of green initiatives and the likelihood of fragmented technological resources transforming into enterprise innovation performance is diminished [44], thus impeding the support of digital transformation in promoting enterprise green governance. Digital transformation plays a pivotal role in advancing green governance among enterprises with robust technological integration.

### 6.3 Further analyses based on the carbon emissions trading pilots

The carbon emissions trading pilot is dedicated to assisting enterprises in achieving the double dividend of their own development and lowering emissions, which aligns with the objectives of corporate green governance. It is a typical market-oriented tool for emission reduction policies [45]. In addition, the pilot program offers a practical means of achieving carbon peaking and neutrality, which can bolster efforts to achieve these objectives. We split the sample into enterprises in pilot carbon emissions trading regions and those in non-pilot regions. The coefficients of digital transformation for companies in the pilot and non-pilot regions are significantly positive, as indicated in Columns (5) and (6) of Table 7. Additionally, the regression coefficient for enterprises in the pilot regions is larger and passes the between-group difference test. It can be inferred that digital transformation fosters green governance in enterprises in both the pilot and non-pilot areas, with a greater contribution from digital transformation in the pilot areas.

The underlying reason is that the carbon trading mechanism mainly plays the role of “icing on the cake,” and under the combined effect of cost pressure and emissions reduction benefits, enterprises are more likely to choose to optimize resource allocation, improve production processes, and embrace other ways to improve production efficiency and achieve their emissions reduction targets [46]. For enterprises in pilot regions, this strengthens the operational efficiency path of digital transformation and creates resource conditions that facilitate corporate green governance. In addition, as the disclosure of carbon information has progressively become a prerequisite for enterprises to engage in the carbon market, the transparency of company data has increased in tandem with the digitalization of the enterprise information environment, strengthening green management. Consequently, the benefits of digital transformation for green governance are greater for businesses in the pilot areas.

## 7. Conclusions and policy implications

### 7.1 Conclusions

With the proposal and implementation of China’s carbon peaking and neutrality goals, elevating the standard of environmental governance has progressively emerged as a crucial consideration in the transition and advancement of enterprises toward green and high-quality development. Representing a significant revolution aimed at optimizing enterprise resource allocation and fostering information connectivity, digital transformation offers an opportunity to catalyze the endogenous momentum of green governance.

We selected A-share listed companies from 2012 to 2021 as our sample and employed the entropy power method to create a corporate green governance index system across five dimensions. We systematically investigated the influence of digital transformation on corporate green governance and derived the following conclusions: First, digital transformation can significantly improve corporate green governance. Second, the digital revolution has enhanced corporate green governance by mitigating information asymmetry and enhancing operational efficiency. Third, the positive correlation between the two variables intensifies as an enterprise’s’ strategic aggressiveness increases. Fourth, the effects of digital transitions on green governance vary depending on the region’s participation in the carbon emissions trading pilot program, the development of digital finance, and the level of technological integration.

### 7.2 Policy implications

Drawing from the above conclusions, we present the following recommendations:

First, we should hasten enterprises’ digital transformation process, encourage the extensive integration of digital technology into enterprise green governance, and offer technological assistance to advance the carbon peaking and neutrality objectives. Initially, the government should provide a framework for policy support that integrates green governance with enterprise digitalization strategies, actively encourage businesses to drive digital transformation, and foster the growth of digitalization and greening in concert. To improve green governance, governments should create targeted preferential policies for low-carbon technological R&D to improve green governance. Moreover, given that digital transformation is a clear catalyst for green governance, businesses should accelerate the pace at which they incorporate digital technology into their daily operations and reasonably integrate their digital strategies into green governance activities such as green management, green emissions, and green regulation to exploit the high penetration and versatility of digital technology and continue to unleash the effects of the digital revolution on green governance.

Second, digital transformation can improve operational efficiency and optimize an organization’s information transmission system, providing a practical route to green governance. Given that digital transformation facilitates green governance by reducing information asymmetry, enterprises should dedicate efforts to clearing internal and external information blockages and elevating the standard of environmental information transparency. Internally, enterprises should strengthen green management through information communication, shape a green culture and concepts, and lay a strong foundation for improving green governance. Externally, enterprises should form positive interactions with consumers, investors, and other stakeholders through information feedback, and continuously enhance the level of green governance to deliver improved environmentally friendly goods and services. Additionally, the government must scientifically formulate information disclosure standards, accurately and objectively disclose corporate green information, and ensure the comparability of information. However, because digital transformation promotes green corporate governance by enhancing operational efficiency, it should empower efficient operations must be empowered. Enterprises can use digital transformation to achieve lean management and develop a new model of efficient operational management that can be applied to green activities. Additionally, enterprises should focus on improving the operational efficiency of their assets, mitigate potential resource conflicts between digital transformation and green governance, and increase green investments to achieve optimal resource allocation.

Third, enterprises must be aware of how their strategies are changing and take proactive steps to implement green governance and digital transformation. Enterprises must focus on long-term growth goals to establish a reasonable plan and strategically emphasize the incorporation of digital technology into internal enterprise operations, because they cannot simply seek stability and ignore development trends in the face of intense competition. Compared with enterprises with more aggressive strategies, conservative enterprises lack enthusiasm for new technologies and emerging markets. Although both digital and green transformations involve certain risks, under the constraints of carbon peaking and neutrality goals, enterprises with less aggressive strategies need to shift to green transformation in their production, operation, and service aspects and actively introduce and apply emerging digital technologies to increase their long-term value through partial adjustments of their strategies.

Fourth, it is imperative to foster an accurate interface between digital finance and enterprises, improve technological integration capacities, and create an environment conducive to businesses engaging in carbon emissions trading. Governments should increase the reach and frequency of digital finance and offer support for digital transformation to promote green governance initiatives. This is because a well-developed digital financial environment is an externally beneficial condition for the role of digital transformation. Enterprises should use digital financing to enhance information transparency and resource allocation efficiency. By providing specialized funding and support facilities for green governance, they can pave the way for digital transformation to influence green governance positively. Because strong technological integration capability is an internal advantage for digital transformation to exert green governance effects, enterprises need to flexibly master digital technologies, digest and refine internal technological resources, and understand how digital technologies can be used in the green field. In addition, the enhancement of technological integration capability is a long-term process, and enterprises must increase their investment and accumulation of technological resources, cultivate innovative human capital, and develop competitive advantages. As the carbon emissions trading pilot policy offers incentives for enterprises in the region to engage in green governance, enterprises must implement the corresponding policies under carbon peaking and neutrality goals and respond to external policy tools while paying attention to the internal drive to form a synergy that improves green governance. The public’s demand for going green must increase, necessitating the establishment of a fair and competitive market. Furthermore, the implementation of a national carbon market is crucial to provide enterprises with favorable development opportunities to engage in carbon emissions trading. Strengthening the impact of digital transformation on corporate green governance must be strengthened.

### 7.3 Research limitations and prospects

This study had certain limitations that warrant further research. On the one hand, the green governance of a considerable portion of Chinese enterprises is still in the exploration and enhancement stage, and the evaluation index system of green governance should continue to be enriched in accordance with changes in the actual situation. It is also possible to study how to measure the efficiency of green governance in terms of inputs and outputs to provide a more accurate evaluation of enterprises’ green governance. Additionally, with the emergence of intelligent technology and digital innovation ecology, new paradigms for promoting green governance will continue to expand. However, the conclusions of this study were drawn from a quantitative analysis of large-sample data, which makes it difficult to achieve specific and refined analyses, as in the case studies. Future researchers could adopt a case study approach to explore the evolutionary process and different paradigms of digital transformation to enhance corporate green governance.

## Supporting information

S1 Data (ZIP)
